# Grape Maturity Estimation Using Time-of-Flight and LiDAR Depth Cameras

**DOI:** 10.3390/s24165109

**Published:** 2024-08-07

**Authors:** Mathew Legg, Baden Parr, Genevieve Pascual, Fakhrul Alam

**Affiliations:** 1 Department of Mechanical and Electrical Engineering, Massey University, Auckland 0632, New Zealand; 1badenparr@gmail.com (B.P.);; 2 Department of Electrical & Electronic Engineering, Auckland University of Technology, Auckland 1010, New Zealand; fakhrul.alam@aut.ac.nz

**Keywords:** grapes, maturity, depth cameras, Kinect Azure, Intel L515

## Abstract

This article investigates the potential for using low-cost depth cameras to estimate the maturity of green table grapes after they have been harvested. Time-of-flight (Kinect Azure) and LiDAR (Intel L515) depth cameras were used to capture depth scans of green table grape berries over time. The depth scans of the grapes are distorted due to the diffused scattering of the light emitted from the cameras within the berries. This causes a distance bias where a grape berry appears to be further from the camera than it is. As the grape aged, the shape of the peak corresponding to the grape became increasingly flattened in shape, resulting in an increased distance bias over time. The distance bias variation with time was able to be fitted with an $R^{2}$ value of 0.969 for the Kinect Azure and an average of 0.904 for the Intel L515. This work shows that there is potential to use time-of-flight and LIDAR cameras for estimating grape maturity postharvest in a non-contact and nondestructive manner.

## 1. Introduction

The timing of when grapes ripen can vary and depends on several factors, including climatic conditions [1,2]. Assessing the maturity of grapes is desirable for viticulturists since it helps with the timing of vineyard management tasks and enables the harvest of grapes at a maturity level that enhances grape quality for both wines and table grapes by optimising the levels of sugars, acids, and other flavour-enhancing compounds [3]. It is also desirable to be able to measure grape properties after harvest. For example, the ability to measure grape freshness could reduce postharvest wastage of grapes [4,5]. The water content of grapes is an example of a property related to freshness that may be desirable to measure. Water loss is generally undesirable for most fruits as it leads to shrinkage. However, when making some wines, the grapes are deliberately dehydrated under controlled conditions before fermentation as this can cause desirable flavour changes in the grapes [6,7].

Manual inspection of grape properties usually involves visual inspection, tasting, and weighing of the grapes. However, this can be time consuming and subjective, leading to under-sampling. Methods such as refractometer Brix tests for sugar levels and chemical testing exist. However, these approaches are destructive [8]. Techniques have therefore been developed to allow the automatic estimation of grape maturity in a non-contact manner [9].

One of the main non-contact techniques used to estimate grape ripeness before harvest is computer vision. Grape ripeness has been estimated by analysing the colour of grapes within the visual spectrum using camera images [10,11]. Near-infrared (NIR) spectroscopy is also commonly used for grape ripeness estimation. Certain wavelengths have been shown to correlate with properties such as sugar and water content [12,13,14]. NIR and visual light may be combined with visual light analysis [15,16,17]. Hyperspectral imaging has also been used for grape ripeness estimation [18]. It has advantages over classical NIR in that it combines spectroscopy with traditional imaging, providing spatial information. Machine learning techniques have been used for both colour and hyperspectral imaging [19]. Refer to the review articles in references [2,20,21] for more details on computer vision estimation of the ripeness of grapes. A range of other techniques have been used to estimate grape ripeness properties. For example, optical techniques such as measurement of the fluorescence of grapes have been used to estimate grape ripeness [22,23,24]. Grape firmness has also been measured before harvest using acoustic techniques [25].

Research has also been performed on nondestructive assessment of the maturity/quality of grapes after harvest. However, there appears to be less work in this area compared to preharvest ripeness estimation. Similar techniques to those used for preharvest grape ripeness estimation have also been applied to postharvest maturity measurements. Much of these postharvest works have used computer vision techniques. For example, Bahar et al. [26] investigated the browning of grape pedicels as an indicator of grape deterioration. Cavallo et al. [27] used computer vision and machine learning to grade table grape bunches. Similarly, hyperspectral imaging has been used to estimate table grape quality postharvest [28].

In previous work by the authors [29,30], a range of low-cost depth cameras were used to image grapes. This covered four depth camera technologies: structured light (Kinect V1), active infrared stereoscopy (Intel RealSense D415), time of flight (ToF) (Kinect V2, Kinect Azure, and Samsung Note 10+ smartphone), and LiDAR (Intel RealSense L515). The cameras that used the time taken for light to be reflected from an object (the time-of-flight and LiDAR cameras) were found to produce depth scans of grape berries that were distorted. The shape of each grape berry in the depth map was distorted to form a peak that was centred at the location of the grape berry. There was also a distance bias where the depth scan points corresponding to the berry were located further away from the camera than the grape actually was. This was observed for both green and red grapes. This distortion and distance bias disappeared when the grape was sprayed with paint. This showed that the shape distortion and distance bias were due to diffused scattering within the grape berries [31].

Parr et al. [29,30] raised the question of whether the distortion effect observed for the time-of-flight and LiDAR cameras could be used to estimate grape properties such as maturity. The water content of the grapes will reduce as they mature after harvest. It has been reported that metabolic changes occur in grape berries as they lose water content after harvest [7]. This can include increased sugar content due to a concentration effect. Could water loss change the optic properties such as the refractive index and the transparency of the berries with time? Could such changes in grape properties cause changes in the distortion and distance bias experienced by these time-of-flight and LiDAR depth cameras when imaging grapes as they mature after harvest? There has been no previous work investigating if time-of-flight and LiDAR depth cameras can be used to estimate the maturity of grapes after harvest. However, Sarkar et al. [32] showed that time-of-flight cameras produced depth scans of a harvested apple that were biased to be further away from the time-of-flight camera than the apple actually was. This distance bias increased with time. Neupane et al. [33] also showed similar results for mangoes.

This article is the first work to investigate if low-cost time-of-flight and LiDAR depth cameras can be used for proximal sensing of the maturity of grapes. The structure of the article is as follows. Section 2 outlines the methodology used to capture and analyse time-of-flight and LiDAR camera scans of grapes over several days. Results are presented in Section 3. A discussion is provided in Section 4. The conclusion is then provided in Section 5.

## 2. Methodology

Measurements were made with the Kinect Azure (Redmond, WA. USA) time-of-flight and Intel L515 LiDAR (Santa Clara, CA, USA) depth cameras. These depth cameras were chosen because they represent two different depth scanning technologies (time of flight and LiDAR), which our previous work [29] had shown to produce depth scans of grapes that were distorted and had distance biases. It should be noted that these two cameras produced slightly different distortion patterns when they were used to image grapes. This may be due to the different depth scanning techniques used by these two cameras. Time-of-flight cameras operate by sending out a pulse of light and measuring the time taken for the light to be reflected back to the camera. It simultaneously measures depth from a wide range of angles using depth-sensing pixels. In contrast, a LiDAR sequentially scans through a range of different angles using a moving mirror.

### 2.1. Kinect Azure Time of Flight Camera Measurements

**Figure 1:**
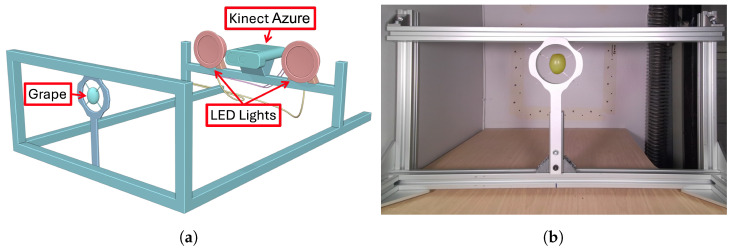
Diagram (**a**) and photo (**b**) of the experimental setup used for Kinect Azure scans of a grape berry over a number of days.

Experiments were first conducted using the Kinect Azure depth camera, which operates using time-of-flight technology. The camera was attached to a frame to ensure it did not move during measurements (see Figure 1). Two LED lights were mounted on either side of the time-of-flight camera to ensure that consistent lighting was maintained over multiple days. A single green table grape was positioned about 360 mm directly in front of the Kinect Azure camera. A holder for mounting the grape berry was laser cut from a piece of Medium-Density Fibreboard (MDF). This holder was painted white and attached to the framing system that held the camera. Needles were used to position the grape at the centre of a circular hole in the holder. It was felt that these needles would hold the grape berry securely but would minimise any potential time-of-flight depth errors that could occur if an object was placed next to the grape, which could cause multi-path reflections. Care was taken to avoid puncturing the skin of the berry too deeply.

**Figure 2:**
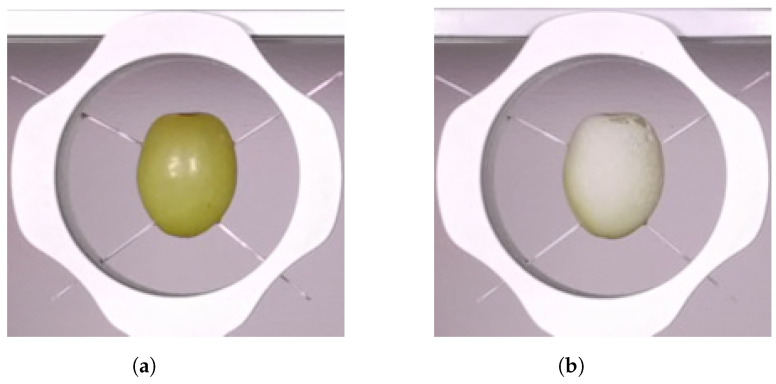
Cropped images of the grape captured by the Kinect Azure’s RGB (red, green, and blue) camera before (**a**) and after (**b**) being sprayed by the coating on Day 1.

Depth and colour images of the grape were captured once a day using the Kinect Azure time-of-flight camera and saved to file. Scans were made each day before and after spraying the grape with the AESUB 3D Scanning Spray (Recklinghausen, Germany). This spray provided an opaque coating over the grape berry, as illustrated in Figure 2. This aimed to stop diffused scattering from within the grape and hence provide a reference scan of the surface of the grape without the shape distortion and distance bias. This spray coating evaporated from the berries after a few hours. This meant that multiple measurements could be made using the same grape over several days, which would not have been possible had a permanent spray coating such as paint been used [29]. Note that the amount of spray used appeared to affect how effective it was at stopping the distortions due to diffused scattering from occurring.

Measurements were stopped after 13 days as the grape had deteriorated and become shrunken by this time. The depth scans were then converted to point clouds and processed in Python 3.12.3 with some plotting performed using MATLAB R2023b. To eliminate noise, isolated scan points were removed by filtering out points whose average distance to their K-nearest neighbours was greater than a specified threshold. Each depth scan was then cropped to include only the scan points corresponding to the grape’s surface. Analysis was then made to see how the shape of the grape in the depth maps changed over time.

### 2.2. Intel L515 LiDAR Camera Measurements

**Figure 3:**
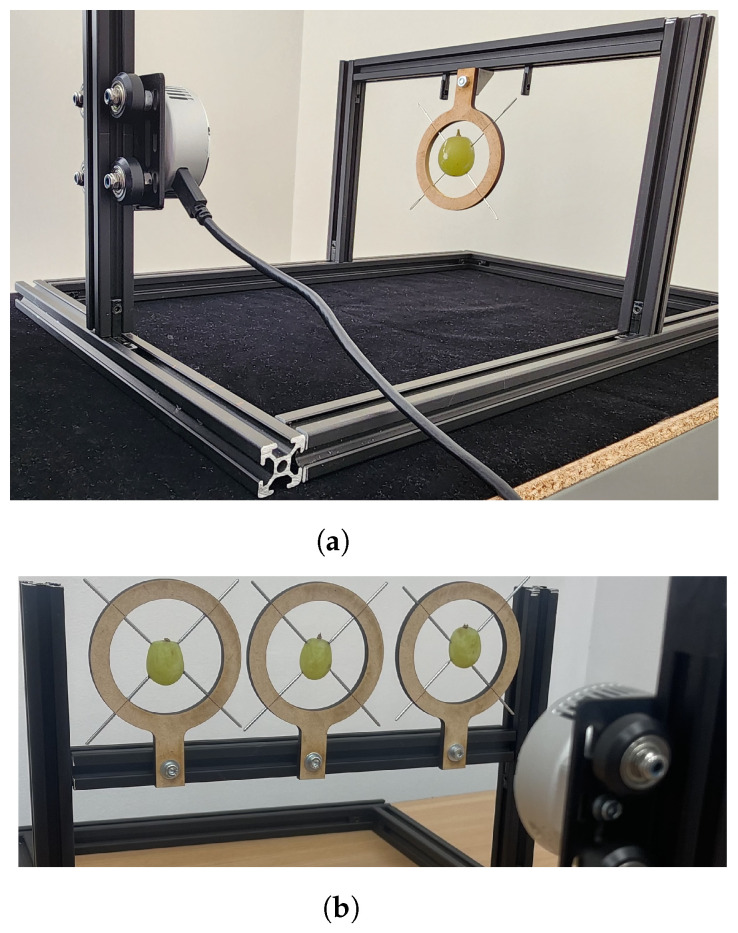
Photos of the experimental setup used to capture depth scans of a single grape (**a**) and multiple grapes (**b**) using the Intel L515 depth camera.

Measurements were also made with the Intel L515 LiDAR depth camera using a similar setup to that of the Kinect Azure. Measurements were made over several days using this depth camera with a single grape berry positioned directly in front of the depth camera at a distance of about 240 and 250 mm (see Figure 3a). Unlike the case for the Kinect Azure measurements, the pedicel was not removed from the grape berry. Depth scans of the grape were made each day for several days. The grape was sprayed on the first and last day of measurements with the AESUB 3D Scanning Spray, and scans were obtained.

The Intel L515 was also used to simultaneously capture scans of three grapes from the same bunch over a number of days. The experimental setup used is shown in Figure 3b. The centre grape was positioned directly in front of the camera (along its optical axis) at a distance of about 255 mm. A second grape was positioned 85 mm to the left of the centre grape. The third grape was positioned 93 mm to the right of the centre grape. Having these extra two grapes on each side of the centre grape allowed extra grapes to be scanned but also provided measurements of grapes that were located at an angle to the depth camera’s optical axis. A second set of measurements was subsequently performed using another set of three grapes taken from a different bunch of grapes.

### 2.3. Water Content Loss

Measurements were also made of the weight of grape berries to estimate the rate of water loss from the grapes. The pedicels of the grapes were left attached to the grapes. Measurements were made of the weight of the grapes each day. The temperature and humidity at the time of the measurements were 20.5 ± 1 °C and 73 ± 1%, respectively. The percentage loss in the grape weight was then calculated using

(1) $$W_{p}=\frac{100(Wp-W_{o})}{W_{o}},$$

where *W* is the weight of the grape on a particular day and $W_{o}$ is the weight on Day 1. Note that different grapes were used for these daily weight loss measurements from those used in the depth scan measurements. This was because it was desirable not to move the grapes during the depth scan measurements.

### 2.4. Ruby and Sapphire Spheres

**Figure 4:**
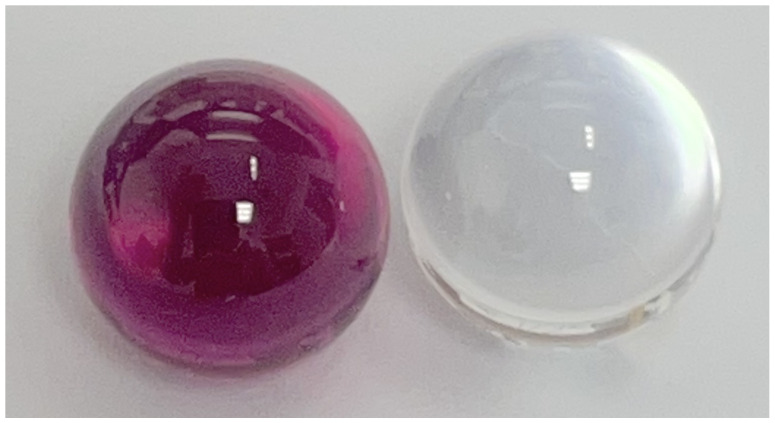
Ruby and sapphire spheres used for comparison with grapes.

Time-of-flight and LiDAR cameras produced distortions and a distance bias when they were used to image grapes. This effect is caused by the fact that the grapes are translucent. There could be a range of factors that may influence this effect. This could include the distance of the grapes from the camera, the grape’s size, and its optical properties such as refractive index and transparency. A challenge when using grapes for these measurements is that grapes are relatively complex, being variable in shape and composition and not having known optical properties. It would therefore be desirable to perform basic measurements using translucent objects that are less complex and have known optical properties. Initial measurements were therefore made with the Intel L515 LiDAR using 9.525 mm diameter ruby and sapphire spheres, which are shown in Figure 4. These were uncoated optical ball lenses with refractive indexes of 1.77 from Edmund Optics (Barrington, NJ, USA). These were placed on a rod with a hole drilled in the top when capturing depth scans. The initial results are presented in Section 3.4.

## 3. Results

### 3.1. Kinect Azure Time of Flight Camera Results

**Figure 5:**
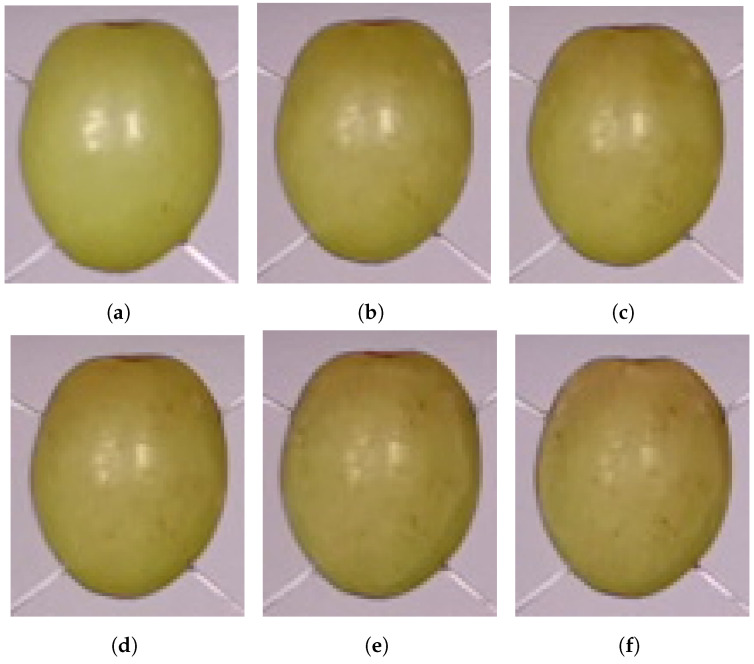
Cropped images of the grape captured by the Kinect Azure’s RGB camera on (**a**) Days 1, (**b**) Days 3, (**c**) Day 5, (**d**) Day 7, (**e**) Day 9, and (**f**) Day 11.

Figure 5 shows cropped colour photos of a grape captured on consecutive days by the Kinect Azure’s RGB camera. It can be seen that the colour of the grape changes over time as the grape matures. This could be used to estimate the ageing of grapes [11]. However, a complication that could occur is that different grapes might naturally have different colouring for the same maturity level, which could lead to errors.

**Figure 6:**
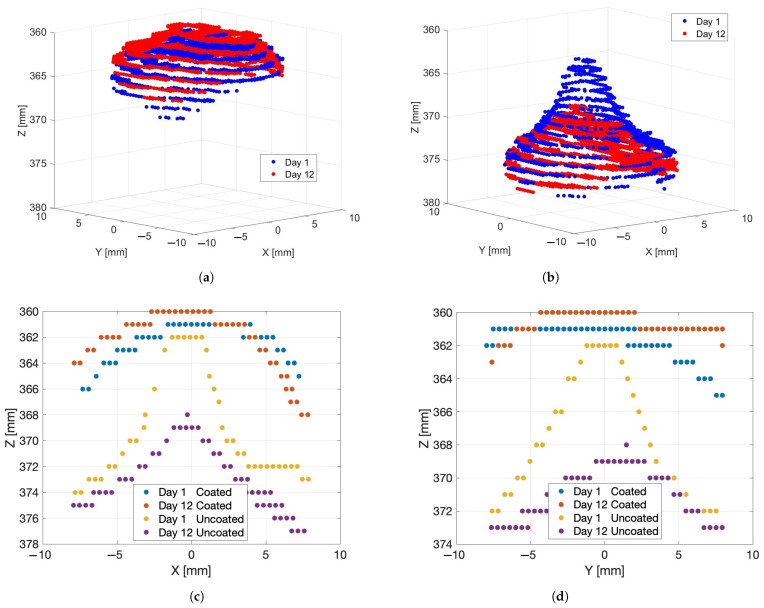
Plot (**a**) shows Kinect Azure scans made of a grape on Day 1 and Day 12 after an opaque coating was sprayed onto the surface of the grape. Plot (**b**) shows the corresponding scans of the grape without any coating. Plots (**c**,**d**) show cross sections through the scans in the *X* and *Y* axes directions. Note that the *Z* axis is the depth or distance from the camera.

Figure 6a shows cropped depth scans of the grape berry captured by the Kinect Azure’s depth camera made on Day 1 and Day 12 after the grape had been sprayed with the AESUB 3D Scanning Spray. The front surface of the grape in the depth scans shows up at a depth of about 360 mm, which was the correct distance between the grape and the camera. The depth scans also reproduce the rounded front surface of the berry. There is relatively little difference between the scans made on Day 1 and Day 12 when the spray is used. This indicates that there has been relatively little change in the shape of the grape over the duration of the measurements.

Figure 6b shows the corresponding depth scans of the grape without any coating on Day 1 and Day 12. Slices through these scans with and without the spray in the X and Y axis directions are shown in Figure 6c,d. It can be seen that the time-of-flight depth scans of the grape without the spray coating are distorted to form peaks centred at the location of the grape. Additionally, there is a depth bias making the grape appear further away from the camera than it actually was. We can see that the height of the peak is smaller (has more distance bias) on Day 12 compared to Day 1. The fact that the distortion and distance bias disappear when the grape is sprayed with an opaque coating shows that the phenomena are caused by diffused scattering of the light emitted by the time-of-flight camera within the berry.

The change in distance between the depth camera and the grape appears relatively small based on the scans of the grape made with the opaque coating on Days 1 and 12 (see Figure 6a). The distance bias is significantly larger than this measured distance change between the camera and the grape. This indicates that the change in the distance bias with time was due mainly to changes in the optical properties of the grape over time rather than being due only to a change in the shape of the grape.

**Figure 7:**
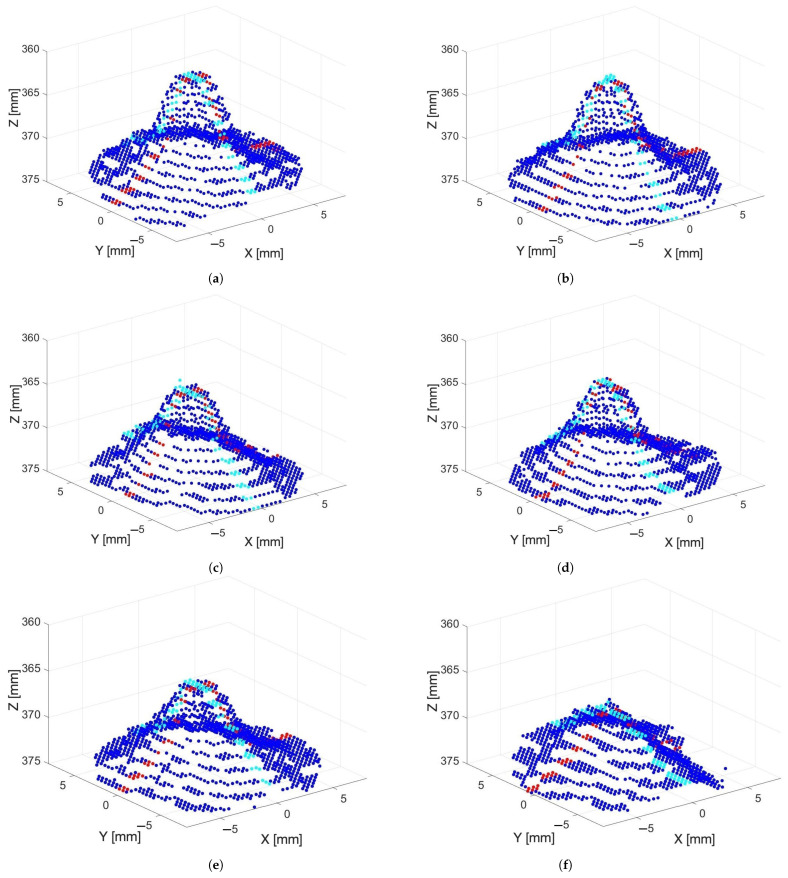
Kinect Azure depth scans of the grape berry captured on (**a**) Day 1, (**b**) Day 3, (**c**) Day 5, (**d**) Day 7, (**e**) Day 9, and (**f**) Day 11 without any spray coating. The red and cyan depth scan points show the cross sections in the *X* and *Y* axes directions that are plotted in Figure 6c,d.

Figure 7 shows example point clouds captured without using the spray coating on Days 1, 3, 5, 7, 9, and 11. It can be seen that the height of the peak steadily reduces over time, though the distance of the base of the peak from the camera appears to remain about the same.

**Figure 8:**
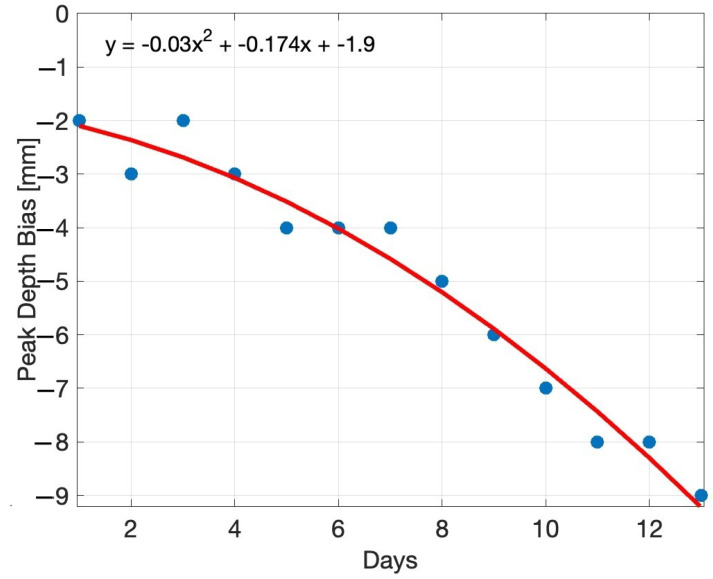
Plot shows the increasing depth error (distance bias) of the Kinect Azure time-of-flight depth scans of the grape over time. This is the error in the depth map for the peak corresponding to the grape relative to the actual distance (360 mm) between the grape and the camera.

Figure 8 shows a plot of the reduction in the depth peak height with ageing. It can be seen that the distance bias *y* of the peak corresponding to the grape in the *z*-axis direction in mm roughly follows a curve described by

(2) $$y=0.03t^{2}-0.17t-1.90,$$

where *t* is time in days. The $R^{2}$ value of the fit is 0.969.

### 3.2. Intel L515 LiDAR Camera Results

**Figure 9:**
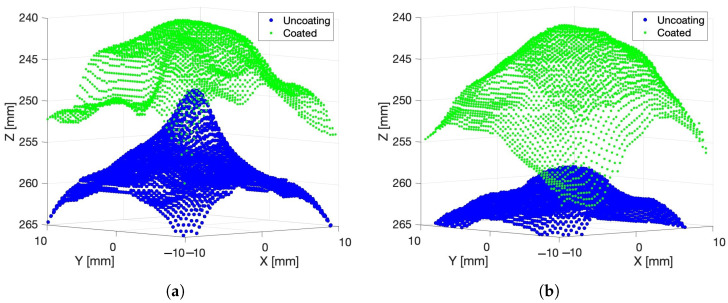
Plots showing the L515 LiDAR scans of a grape located about 240 mm in front of the depth camera on (**a**) Day 1 and (**b**) Day 12 before and after being sprayed with an opaque coating.

Figure 9 shows scans of the grape captured by the Intel L515 LiDAR before and after it had been coated with the AESUB 3D Scanning Spray on Days 1 and 12. It can be seen that with the opaque coating there is relatively little change in scans between Days 1 and 12. This shows that the distance between the depth camera and the surface of the grape facing the depth camera has not changed significantly during the period that the experiments were performed. However, without the opaque coating, the depth scan of the grape is distorted to form a peak and there is a distance bias, which is larger for Day 12 than Day 1. This indicates that the change in the depth bias is due to changes in the optical properties of the grape over time. These results are consistent with those observed for the Kinect Azure.

**Figure 10:**
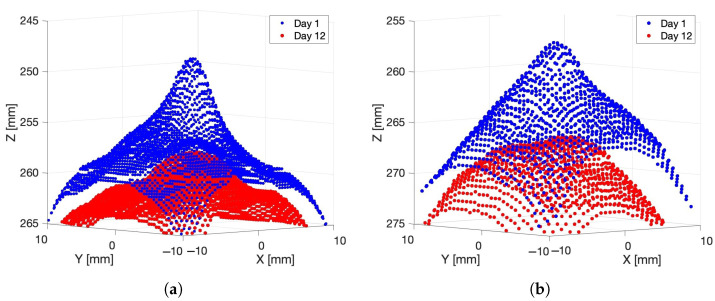
Plot showing the 3D scans captured by the Intel L515 LiDAR of three grapes located (**a**) 240 mm and (**b**) 250 mm, respectively, from the depth camera on Days 1 and 12.

Figure 10a shows example Intel L515 LiDAR depth scans of the grape at about 240 mm from the depth camera, which were made on Days 1 and 12. It can be seen that without any opaque coating over the grape, there is a reduction in peak height over the 12 days. Similar results are observed for the grape positioned at about 250 mm distance from the depth camera (see Figure 10b).

**Figure 11:**
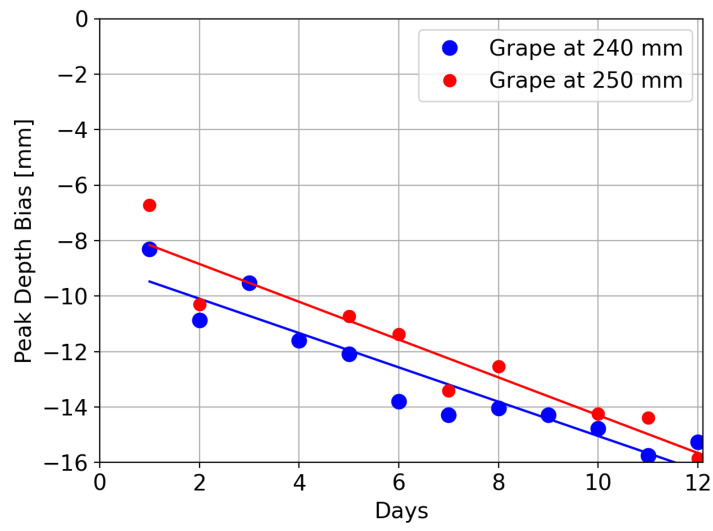
Plots showing the depth error (distance bias) of the Intel L515 LiDAR depth scans of grapes over time.

Figure 11 shows the changes with time in the depth bias of the peak corresponding to the grape, which was positioned about 240 mm from the depth camera. This shows an increase in the distance bias *y* in mm with time *t* in days. A linear fit through the data of

(3) y=−0.62t−8.9,

has an $R^{2}$ value of 0.88. Also shown in Figure 11 are results for a grape held at 250 mm. A linear fit through the data for this grape gives

(4) y=−0.68t−7.48,

which has a $R^{2}$ value of 0.90.

**Figure 12:**
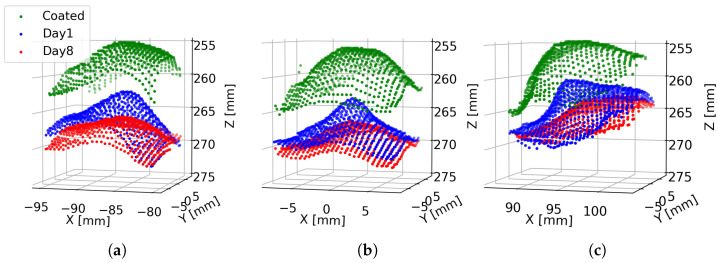
Plot showing the 3D scans captured by the Intel L515 LiDAR of the three grapes shown in Figure 3b. Plots (**a**–**c**) respectively correspond to the left, centre, and right grapes as viewed from the camera. For each grape, a scan is shown where the grape was sprayed with an opaque coating, as well as scans made on Days 1 and 8 without the coating.

Measurements were also made using the Intel L515 LiDAR of three grapes taken from the same bunch (“Bunch 1”). A photo of the experimental setup is shown in Figure 3b. These measurements were repeated using three grapes taken from another grape bunch (“Bunch 2”). Figure 12 shows Intel L515 LiDAR scans captured of the three grapes from “Bunch 1”. Scans are shown for each grape for Days 1 and 8. Additionally, a scan is shown for each grape after it has been sprayed with AESUB 3D Scanning Spray to provide an opaque coating. As has been shown above, it can be seen that without the opaque coating, the peaks experience a shape distortion and a distance bias that increases with time. However, we can also see that the shape corresponding to a grape is different depending on where the grape is located relative to the optic axis of the depth camera. The grape at the centre (positioned on the optic axis) has a symmetric shape around the *Z*-axis, and the peak appears to be located directly behind the grape location. However, for the grapes on either side, the shape of the peaks appears to ”point” towards the depth camera. That is, the peak corresponding to a grape appears to be symmetric around a line passing from the depth camera through the grape’s location.

**Figure 13:**
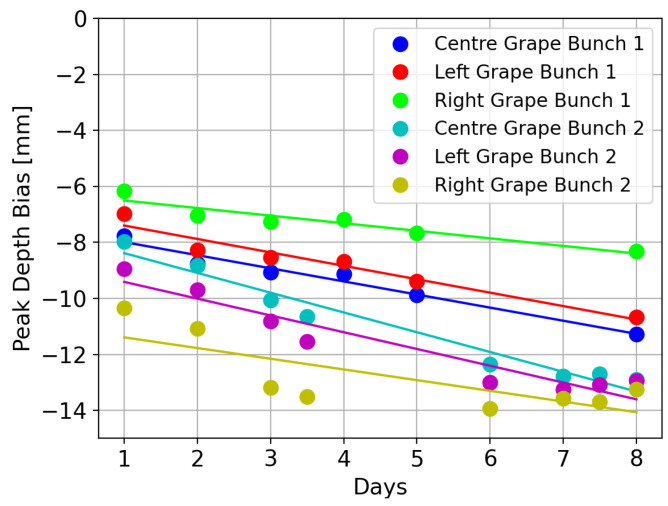
Plots showing the distance bias measurements over time for the three grapes from two different bunches.

Figure 13 shows measurements of the distance bias for the grapes from Bunch 1 and 2 over several days. This distance bias was calculated by measuring the distance in the *Z*-axis direction between the peak in the scan of each grape made with the opaque coating and those made on each day without the coating. It can be seen that for all the grapes, there is an increase in distance bias with time. Fitted lines through the data with the corresponding $R^{2}$ values are:

(5) $$\begin{array}{cc} \mathrm{Bunch}\;1: & \left\{\begin{array}{cc} y_{L} & =-0.45\;t-7.06\;\;\;\;(R^{2}=0.93)\\ y_{C} & =-0.46\;t-7.57\;\;\;\;(R^{2}=0.94)\\ y_{R} & =-0.25\;t-6.34\;\;\;\;(R^{2}=0.87), \end{array}\right. \end{array}$$

(6) $$\begin{array}{cc} \mathrm{Bunch}\;2: & \left\{\begin{array}{cc} y_{L} & =-0.60\;t-8.81\;\;\;\;(R^{2}=0.92)\\ y_{C} & =-0.71\;t-7.68\;\;\;\;(R^{2}=0.96)\\ y_{R} & =-0.38\;t-11.01\;\;\;(R^{2}=0.59), \end{array}\right. \end{array}$$

where $y_{L}$, $y_{C}$, and $y_{R}$ are the fitted distance biases in mm for the left, centre, and right grapes, respectively, as a function of time *t* in days. The right grape from Bunch 2 has the lowest $R^{2}$ value. Its distance bias seemed to flatten off after Day 3. It appeared that this grape showed a higher decay by the end of the experiments than the other grapes.

More consistent results might be achieved by measuring the distance bias along a line passing through the depth camera and the grape location rather than in the Z-axis direction. This is because the peaks corresponding to the grapes appear to be symmetric around this line.

### 3.3. Water Content Loss Estimation Measurements

**Figure 14:**
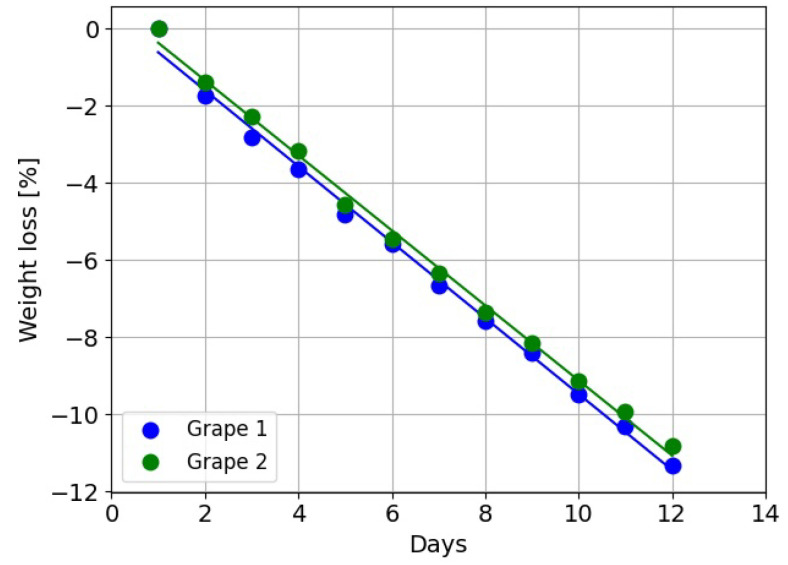
Measured percentage of weight loss of grapes with time. This should correlate with the percentage of water content loss.

Measurements were made of the weight of grapes to estimate the rate of water content loss. Figure 14 shows the percentage reduction in the weight of grapes over time. The percentage weight loss was about 0.98% per day. Note that different grapes were used for these weight loss experiments to those used in the depth scan measurements.

### 3.4. Glass Sphere Measurements

**Figure 15:**
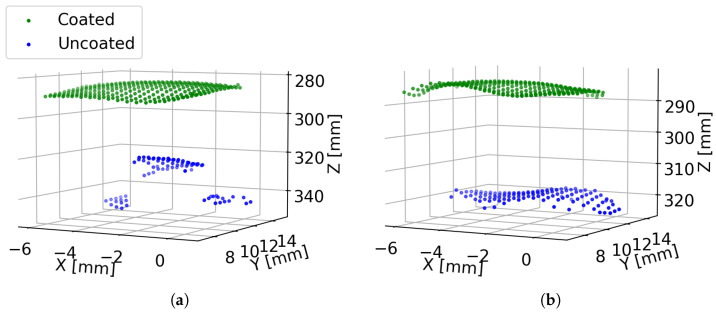
Cropped Intel L515 depth scans of (**a**) ruby and (**b**) sapphire glass spheres before and after being sprayed with an opaque coating.

Depth scan measurements were made of 9.53 mm diameter ruby and sapphire spheres using the Intel L515 LiDAR. This was done to see if these also produced distance biases like the grapes. The spheres were located about 230 mm from the depth camera. These measurements were made with and without the opaque AESUB 3D Scanning Spray. Figure 15 shows the cropped depth scans of these spheres. It was harder to detect the glass spheres without the coating. However, it can be seen that, like the grapes, a distance bias is observed when no opaque coating was used. These distance biases were 37.6 and 35.6 mm, respectively, for the ruby and sapphire spheres.

These initial results show that there may be potential for using translucent materials such as glass spheres for basic measurements to obtain a better understanding of how factors such as diameter and refractive index might affect the distortion and distance bias phenomena for grapes.

## 4. Discussion

This study has examined a limited selection of green table grapes. To determine the repeatability of the results and their relationship with factors such as size and colour, additional measurements are needed on a larger and more diverse sample of grape berries. This includes grapes of a range of varieties, colours, sizes, and shapes. More experiments could also be performed with other translucent objects with known optical properties (such as glass spheres) to better understand what factors affect the shape distortion and distance bias phenomena. Measurements are also needed with the depth cameras at a range of distances and the grapes at a range of angles relative to the optic axis of the depth camera to see how this affects the results.

The grapes used for the experiments were purchased from a supermarket. There was no independent measure of how fresh the grapes were when measurements were started. Time was then used as a measure of reduced freshness. In addition, the grapes were kept at room conditions and the temperature and humidity levels would have varied during and between measurements. In future work, it would be ideal to start measurements before and from the time of harvesting of the grapes. Ideally, independent measurements of grape properties should also be made. An example could be to make Brix refractive index measurements of grapes to obtain the sugar content levels.

More work is needed to investigate if there is a correlation between the changes in shape distortion/distance bias and the reduction in the water content of the grapes with time. Measurements of the weight of grape berries showed a linear reduction in the percentage of water content over time. The Intel L515 depth scan measurements similarly showed linear increases in distance bias over time. This indicates that there might be a correlation between the changes in the depth bias and water content loss. However, there could be other factors associated with ageing that may be influencing the results. Future experiments could include measuring the weight of the grape on the first and last day of the experiment and recording the distance bias that occurred during this period. The relationship between distance bias and weight loss, which is related to water content loss, could then be analysed for a larger number of grapes. Additionally, measurements could be performed while controlling the temperature and humidity level as the grapes mature. Lower temperature and high humidity levels would reduce the rate of water loss and decay of the grapes [34].

The Kinect Azure also showed a nonlinear relationship between distance bias and ageing. The variation in results between the Kinect Azure and Intel L515 may stem from their utilisation of distinct depth camera technologies, namely time of flight and LiDAR, respectively. As previously stated, time-of-flight cameras calculate depth maps by capturing light reflected from a range of angles simultaneously, whereas LiDARs create depth maps using a scanning process. However, for the Kinect Azure experiment, the pedicel had been removed, which was not the case for the Intel 515 measurements. This would be expected to have increased water content loss, which may not have been linear. It may also be that the grape used in the Kinect Azure measurements had a different freshness level when the experiments started (e.g., the grape used could have been more or less aged than those used for the Intel 515 measurements). Additionally, other factors such as changes in temperature and humidity could have affected the rate of water content loss. More experiments are needed in the future with this depth camera capturing scans of grapes over time.

In this work, only the depth information was used from the camera. Future work could investigate combining the colour images with the depth information captured by these depth cameras to make a more robust system. Future work could also investigate the optimal way to automatically measure the shape distortion and distance bias in real time. One option that could be investigated is to use a depth scanning system that does not experience shape distortion and distance bias when scanning grapes, such as the RealSense D415, which uses active infrared stereoscopy rather than time-of-flight or LiDAR methods. By comparing an undistorted scan of grapes with the distorted scan of the time-of-flight or LiDAR depth camera, the actual distance bias might be able to be estimated automatically in real time without any opaque spray.

## 5. Conclusions

In previous work by the authors [29,30], it was shown that time-of-flight and LiDAR depth cameras produced scans of grapes that are distorted and have distance biases. Each grape shows up in the depth scan as a peak that is centred at the grape location rather than reproducing the correct shape of the grape. Additionally, the peak appears at a distance in the depth scan that is greater than the actual distance between the grape and the depth camera. This study is the first to explore the evolution of depth scans obtained from time-of-flight and LiDAR depth cameras as grapes age post-harvest. In particular, it looks at how the shape distortions and distance biases of the depth scan of grapes captured by these depth cameras change with the ageing of the grapes.

Experiments were initially taken using a Kinect Azure time-of-flight camera. A single grape with its pedicel removed was positioned about 360 mm from the depth camera and was held in place using needles on a framing system. The time-of-flight camera was used to capture depth and colour scans of the grape each day. The grape appears in the depth scan as a peak centred at the location of the grape, with the peak appearing further from the camera than the grape actually was (a distance bias). By spraying the grape with an opaque coating, this shape distortion and distance bias were removed, showing that it is due to diffused scatting of the light emitted by the time-of-flight depth camera. Measurements were made of the grape for 12 days. It was observed that without the spray coating, the shape of the peak corresponding to the grape berry changes over time. The distance bias increased with time. This distance bias was fitted by a quadratic curve with an $R^{2}$ value of 0.969.

Measurements were also repeated using an Intel L515 LiDAR depth camera to capture depth scans of grape berries. Unlike the case for the Kinect Azure, the grapes used for the L515 LiDAR measurements did not have their pedicels removed. Measurements were made with a grape berry at about 240 and 250 mm directly in front of the depth camera. Both sets of experiments produced distance biases that increased with time. They were fitted using a straight line with $R^{2}$ values of 0.88 and 0.90, respectively. Additional measurements were made of six grapes (three grapes at a time) from two different bunches of grapes. These also showed a linear increase in distance bias with time with $R^{2}$ values of 0.93, 0.94, 0.87, 0.92, 0.96, and 0.59. The peaks corresponding to the grapes were symmetric around a line passing through the camera and the location of the grape. This indicates that if the grape is not located near the depth camera’s optical axis, improved accuracy in the distance bias measurement would be obtained by measuring it along this line rather than just in the *Z*-axis direction.

Both the Kinect Azure and Intel L515 LiDAR showed increased distance bias of the depth scans of grapes as they aged after harvest. These results are consistent with the results reported by Sarkar et al. [32] and Neupane et al. [33] for apples and mangoes. These results indicate that there may be potential for using shape distortion and distance bias in time-of-flight and LiDAR depth cameras for estimating the maturity of grapes after harvest. This has the potential to provide a new independent way of proximally sensing grape maturity in real time. However, more measurements are needed with a larger number of grapes and grapes of different varieties to investigate how repeatable the results are. Work is also needed to understand what factors influence the distance bias and shape distortion for the depth cameras.

## Data Availability

The data presented in this study are available on request from the corresponding author.
